# Changes in the Gut Microbiome Contribute to the Development of Behcet’s Disease *via* Adjuvant Effects

**DOI:** 10.3389/fcell.2021.716760

**Published:** 2021-09-08

**Authors:** Qingfeng Wang, Shenglan Yi, Guannan Su, Ziyu Du, Su Pan, Xinyue Huang, Qingfeng Cao, Gangxiang Yuan, Aize Kijlstra, Peizeng Yang

**Affiliations:** ^1^The First Affiliated Hospital of Chongqing Medical University, Chongqing Key Laboratory of Ophthalmology, Chongqing Eye Institute, Chongqing Branch of National Clinical Research Center for Ocular Diseases, Chongqing, China; ^2^University Eye Clinic Maastricht, Maastricht, Netherlands

**Keywords:** gut microbiome, autoimmune disease, Behcet’s disease, fecal transplantation, T cells, neutrophils

## Abstract

Behcet’s disease (BD) is associated with considerable gut microbiome changes. However, it still remains unknown how the composition of the gut microbiome exactly affects the development of this disease. In this study, transplantation of stool samples from patients with active ocular BD to mice *via* oral gavage was performed. This resulted in decreases of three short chain fatty acids (SCFAs) including butyric acid, propionic acid and valeric acid in the feces of the BD-recipient group. Intestinal barrier integrity of mice receiving BD feces was damaged as shown by a decreased expression of tight junction proteins and was associated with the release of Lipopolysaccharides (LPS) in the circulation. The mice also showed a higher frequency of splenic neutrophils as well as an enrichment of genes associated with innate immune responses in the neutrophils and CD4 + T cells as identified by single cell RNA sequencing. Analysis of neutrophils and T cells functions in these mice showed an enhanced mesenteric lymph node and splenic Th1 and Th17 cell differentiation in association with activation of neutrophils. Transplantation of BD feces to mice and subsequent induction of experimental uveitis (EAU) or encephalomyelitis (EAE) led to an exacerbation of disease in both models, suggesting a microbial adjuvant effect. These findings suggest that the gut microbiome may regulate an autoimmune response *via* adjuvant effects including increased gut permeability and enhancement of innate immunity.

## Introduction

Behcet’s disease (BD) is a chronic, multisystemic inflammatory disorder (Zeidan et al., 2016), characterized by recurrent oral and genital ulcers, skin lesions, as well as a sight-threatening intraocular inflammation called panuveitis (Takeuchi et al., 2015). BD is thought to share both autoimmune and autoinflammatory disease features caused by an aberrant population of Th1 and Th17 cells in combination with hyper-activated neutrophils (Yamashita, 1997; Nanke et al., 2008). The exact etiology of the disease is however not yet clear.

Changes in the gut microbiome composition are thought to contribute to the development of various immune and infectious diseases (Bevins and Salzman, 2011; de Oliveira et al., 2017; Russler-Germain et al., 2017; Domingue et al., 2020). Dysbiosis of the gut microbiota has been found in rheumatoid arthritis (RA) and type 1 diabetes (T1D) using metagenomic sequence (MGS) and 16S rDNA gene sequence analysis (Zhang et al., 2015; Vatanen et al., 2018). Studies dealing with the pathogenesis of autoimmunity have suggested that the composition of the gut microbiota plays a crucial role in the development of auto-reactive lymphocytes and the recruitment of neutrophils (Littman and Rudensky, 2010). Some opportunistic intestinal pathogens have been shown to produce the effector pathogen-associated molecular pattern (PAMP) and microbe-associated molecular pattern (MAMP) molecules which can trigger an inflammatory response *via* host cell receptors such as toll like receptors (TLRs) (Fraiture and Brunner, 2014). An impaired gut barrier function caused by metabolites released from the gut microbiota may facilitate this response (Wang et al., 2012; Furusawa et al., 2013).

We recently started investigating the role of the gut microbiome in the development of ocular BD (Consolandi et al., 2015; Ye et al., 2018). Recent studies provided strong evidence that the gut microbiome contains triggers and/or amplification signals for autoreactive T cells that can drive spontaneous uveitis in mice (Horai et al., 2015). These findings may also have implications for the contribution of the gut microbiome to the etiology of human uveitis. However, the mechanisms underlying the contribution of the gut microbiome to the development of clinical uveitis is still unclear. In the present study, we provide evidence that the BD gut microbiome may contribute to the development of intraocular inflammation *via* a complex mechanism including altered gut permeability and immunological adjuvant effects.

## Materials and Methods

### Study Participants

For fecal transplantation, five active BD patients along with 5 sex- and age-matched healthy controls were recruited for this study. The active patients included for the study showed active ocular inflammation and had stopped taking immunosuppressive medicines except topical corticosteroids and cycloplegics for at least 1 month, prior to sampling when they visited our clinic. Healthy controls with diabetes, cardiovascular diseases, systemic disease and other inflammatory diseases were excluded. Additionally, the subjects enrolled for sample collection had not received antibiotics or probiotics for at least 1 month (BD patients are generally not treated with antibiotics). Stool samples from five untreated active BD patients and five healthy controls (the metadata of samples for fecal transplantation are shown in Supplementary Table 1) were used to colonize mice. Diagnosis of BD was based on the diagnostic criteria of the international study group for BD (Criteria for diagnosis of Behcet’s disease, 1990). Active BD was defined according to the presence of active intraocular inflammation. The study was approved by the Ethics Committee of Chongqing Medical University. Signed informed consent was obtained from all participants at the beginning of the study. All procedures were performed in accordance with the Declaration of Helsinki.

### Fecal Microbiome Transplantation in Mice

A fecal sample from each donor was resuspended in sterile PBS to a final concentration of 200 mg/ml and then equal volumes of five donor suspensions were pooled. The suspensions were divided in 1 ml portions per centrifuge tube and stored at −80°C until use. The same batch of fecal samples from BD patients and controls was used for the entire study. Each mouse was orally administered with 200 μl of the pooled fecal suspension once a day for 1 week as described in our previous study after being treated with an antibiotic cocktail containing ampicillin (1 mg/ml), neomycin (1 mg/ml), metronidazole (1 mg/ml), and vancomycin (0.5 mg/ml) (all purchased from Sigma-Aldrich) for 3 weeks (Ye et al., 2018).

### Autoimmune Animal Models: Induction, Clinical and Histological Assessment

B10.RIII mice and C57BL/6 mice were purchased from Jackson Laboratory (Bar Harbor, ME, United States) and maintained under specific pathogen free (SPF) conditions. EAU induction was induced in B10.RIII mice using 25 μg of interphotoreceptor retinoid binding protein (IRBP)_161__–__180_, which is half the dose of peptide for experimental uveitis (EAU) induction as used in earlier studies, in complete Freund’s adjuvant (CFA) (Sigma-Aldrich, St. Louis, MO, United States) supplemented with 1.0 mg/ml *Mycobacterium tuberculosis* strain (MTB) (Jiang et al., 2008). For experimental encephalomyelitis (EAE) induction, female C57BL/6 mice were immunized with 100 μg of myelin Oligodendrocyte Glycoprotein (MOG) 35–55 (half dose of peptide for EAE induction before) emulsified in CFA. 200 ng of pertussis toxin (List Biological, Campbell, CA, United States) was injected intraperitoneally (i.p.) 0 and 48 h later (Constantinescu et al., 2011). Low doses of peptides were used to obtain a milder form of the disease so as to enable experiments showing a worsening effect of our experimental conditions on both the EAU as well as the EAE model. To obtain clinical and histological scores of the EAU model, animals were scored at day 14 as described previously (Cortes et al., 2008). The clinical score and body weight of EAE mice was measured starting at 16 days after immunization (Badawi et al., 2012). The animal study was approved by the Ethics Committee of the First Affiliated Hospital of Chongqing Medical University.

### Isolation of Lymphocytes and Cytokine Secretion Assays

Lymphocytes from mouse spleen were filtered with cell strainers and purified by mouse Ficoll–Hypaque density gradient centrifugation. The cells were cultured in culture medium consisting of RPMI medium 1640, 100 U/ml penicillin/streptomycin and 10% fetal bovine serum (Invitrogen, CA, United States). For cytokine secretion assays, cells (5 × 10^5^/500 μl) were seeded into each well of a 48-well plate. The supernatants of lymphocytes after stimulation with peptides were collected after 3 days. The production of IFN-γ and IL-17 in the supernatants was quantified using Duoset ELISA development kits (R&D Systems, MN, United States) according to the manufacturer’s instructions. For statistical analysis, concentrations below the detection limit were converted to a value of 50% of the lowest point of the calibration curve (de Jager et al., 2005).

### Assessment of Intestinal Barrier Function and LPS in Serum

The colon and serum samples were collected from mice at day 7 after fecal transplantation. Three tight junction proteins were detected by real-time PCR as described above and Western Blotting (WB) analysis. Protein was extracted from colon tissues by radio immunoprecipitation assay (RIPA) lysis buffer (Beyotime, Shanghai, China) including 1% protease inhibitor (Beyotime). WB analysis was performed as described previously (Yang et al., 2014). Bands were analyzed using Image J software, version 1.43. Analysis was normalized against β-actin. Specific primary antibodies used included: Claudin-1 (1/1,000, ImmunoWay, United States), Claudin-4 (1/1,000, ImmunoWay, United States), Occludin (1/1,000, ImmunoWay, United States) and β-actin (1/10,000, ABclonal, United States). The level of Lipopolysaccharide (LPS) in serum was quantified using an Instant ELISA Kit for LPS in accordance with the manufacturer instructions (USCN, Wuhan, China).

### Neutrophil Isolation and Neutrophil Extracellular Traps Detection

Neutrophils were isolated from mouse spleen, filtered with cell strainers and purified by Ficoll–Hypaque density gradient centrifugation. After centrifugation, red blood cell (RBC) lysis was performed to obtain pure neutrophils (Coquery et al., 2012). For mesenteric lymph node, we isolated total cells from tissues and filtered by cell strainers. For eye tissue, we dissected the retina from eye balls after removing the cornea, sclera and choroid, and then we isolated total cells from retina by filtering through cell strainers. 1 × 10^6^ cells from these tissues were seeded into each well of a 48-well plate and stimulated with 100 nM phorbol 12-myristate 13-acetate (PMA) for 4 h at 37°C. After stimulation with PMA, slides with neutrophils were washed by PBS and fixed with 4% paraformaldehyde in PBS. Blocking was performed with 10% normal goat serum. Neutrophil extracellular traps (NETs) were detected with rabbit anti-NE (Abcam, Cambridge, MA, United States) and rabbit anti-MPO (Abcam, Cambridge, MA, United States) in 10% normal goat serum. Slides were incubated with goat anti-rabbit IgG secondary antibody (Alexa Fluor 555, ImmunoWay, Plano, TX, United States) and (FITC, ImmunoWay, Plano, TX, United States) in PBS. Images were obtained with a confocal microscope (Nikon, Japan). The supernatants of neutrophil cultures after stimulation were collected for NETs secretion assays. The levels of NE and MPO were quantified in the supernatants of neutrophils using Duoset ELISA development kits (R&D Systems, MN, United States) in accordance with the manufacturer instructions. For co-culture experiments, 1 × 10^6^ lymphocytes were seeded into each well of a 48-well plate and co-cultured with 500 μl neutrophil culture supernatants and 1 μl cell activation cocktail with Brefeldin A for 6 h before flow cytometry.

### Single Cell RNA Sequencing of Splenic Cells

Splenic cells were isolated from mice at day 7 after fecal transplantation. Before loading onto chromium microfluidic chips, red blood cells (RBCs) were removed from the splenic cells by RBC lysis. Sequencing was performed with Illumina (HiSeq 2000) according to the manufacturer’s instructions (Illumina). In the quality control of the raw reads, low-quality reads (the average quality per base drops below 10), trailing low quality or N bases (below quality 3), adapters and drop reads below 26 bases long were removed from raw reads by fastq. Then the raw reads were demultiplexed and mapped to the reference genome by 10X Genomics Cell Ranger pipeline^1^ using default parameters. The SingleR^2^ and Seurat^3^ packages were performed to cluster and annotate cell populations and types. After annotating these cells populations, functional enrichment analysis of differential genes in the different cell types was performed by Gene Ontology (GO) and Kyoto Encyclopedia of Genes and Genomes (KEGG) analysis (Ashburner et al., 2000; Kanehisa and Goto, 2000).

### Flow Cytometry, Reagents and Antibodies

For flow cytometry, cells were isolated from tissues as described before and seeded into each well of a 48-well plate and treated with 1 μl cell activation cocktail with Brefeldin A (Biolegend, San Diego, CA, United States) for 6 h at 37°C. Then cells were washed, fixed and permeabilized using fixation buffer (Biolegend, San Diego, CA, United States) and permeabilization buffer (Biolegend, San Diego, CA, United States) according to the manufacturer’s instructions. The cells were stained with fluorescent antibodies including anti-mouse CD4-APC, anti-mouse IFN-γ-PE-Cy7 and anti-mouse IL-17-PE (all antibodies were purchased from Biolegend, San Diego, CA, United States) for 20 min.

### Real-Time PCR

Total RNA was isolated by TRIzol reagent (Invitrogen, Carlsbad, CA, United States) from PBMCs and colon tissue. The PrimeScript RT kit (Takara Biotechnology, Dalian, China) was used to reverse the extracted RNA into complementary DNA. The ABI Prism 7500 system on the SYBR Premix (BIO-RAD, CA, United States) was used to detect and analyze the expression. The relative expression of target genes was quantified by using the 2^–ΔΔCt^ method with β-actin as the internal reference. The sequences of target genes and β-actin PCR primer pairs are shown in Supplementary Table 2.

### 16S rDNA Gene Sequence Analysis

DNA was extracted from the mice fecal pellets using the QIAamp Fast DNA Stool Mini Kit (Qiagen, Hilden, Germany) according to the manufacturer’s instructions and was subjected to amplification of polymerase chain reaction (PCR) using primers directed at hypervariable region 3–4 (V3–V4) of the 16S rRNA gene (341F and 806R). The PCR products were quantified using Qubit (Invitrogen, Carlsbad, CA, United States). The resulting raw reads matched to sequences spanning the entire V3–V4 amplicon using PANDAseq. The annotation of bacteria was performed by RDP Classifier (version 2.2). The differential abundance of bacteria between groups was analyzed by Wilcoxon test. *P*-value was corrected by False Discovery Rate (FDR). LEfSe analysis was used to explain the features of microbiome composition between the BD and healthy control group. According to the normalized OTU abundance table, PCoA was applied to visualize similarities or dissimilarities of the microbiota of samples in the BD-recipient mice and healthy controls-recipient mice groups and displayed by QIIME2 and ggplot2 package. In brief, a distance matrix of unweighted Unifrac in the samples was transformed to a new set of orthogonal axes. The maximum variation factor was demonstrated by first principal coordinate and the second maximum one by the second principal coordinate. Shannon index was applied to the difference of microbial diversity between the BD-recipient mice with and healthy controls-recipient mice groups. GC-MS analysis was performed using an Agilent 7890B gas chromatograph system coupled with a Agilent 5977B mass spectrometer. This analysis was performed at the Shanghai Biotree Biomedical Technology Co., Ltd., (Shanghai, China).

### Statistical Analyses

The results were analyzed by GraphPad Prism V 7.0. The statistical significance between two independent groups was analyzed with the Mann–Whitney *U* test. The data are shown as mean ± SEM. A *p*-value less than 0.05 was considered as statistically significant.

## Results

### Fecal Transplantation of Stool Samples Obtained From Active BD Patients to Mice

**FIGURE 1:**
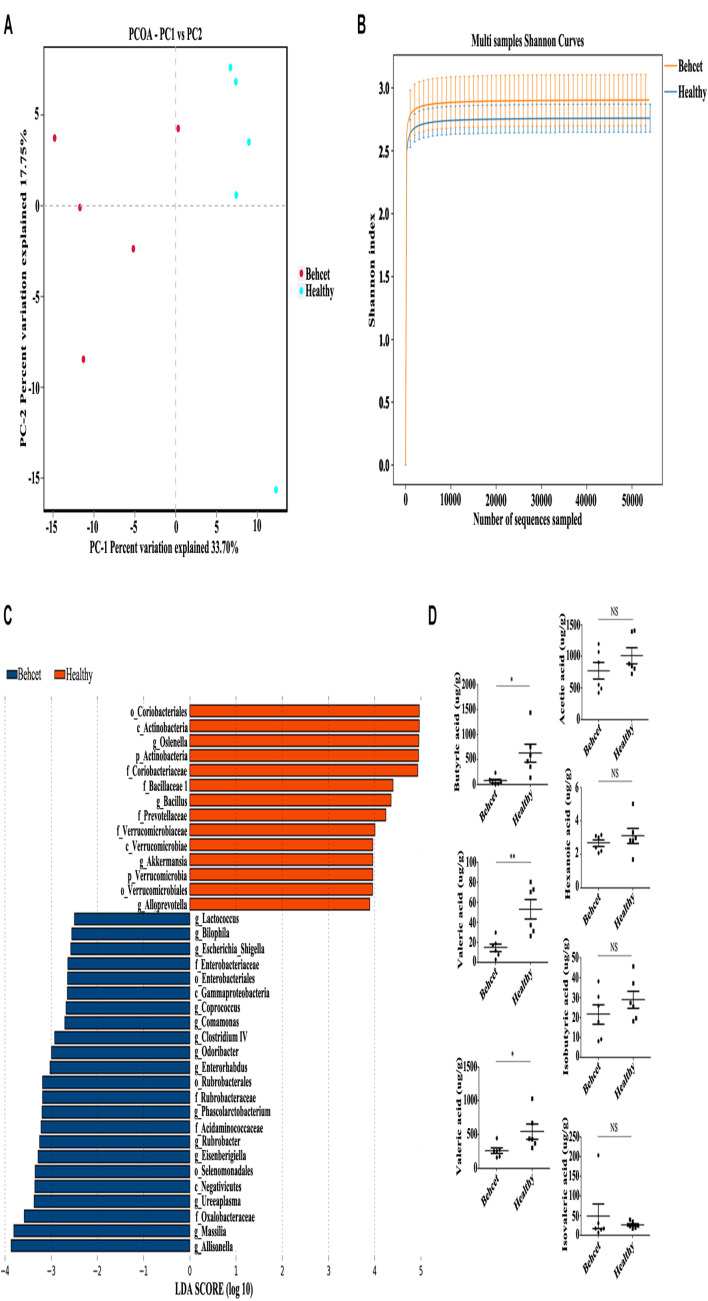
The relative abundances of gut microbiota at genus and species levels and fecal SCFAs comparing the BD-recipient group with the healthy controls-recipient group. **(A)** PCoA analysis of BD-recipient and healthy controls-recipient mice. *n* = 5 for each group; **(B)** Shannon index analysis comparing the BD-recipient and healthy controls-recipient group. *n* = 5 for each group; **(C)** Histogram of the LDA scores computed for taxa differentially abundant between BD-recipient mice and healthy controls-recipient mice. **(D)** The concentrations of SCFAs in the feces comparing BD-recipient mice and healthy controls-recipient mice. **P < 0.01, *P < 0.05, NS, not significant. *n* = 6 for each group.

To study the role of the gut microbiome in the development of ocular Behcet’s disease, we used a previously established method of transplanting pooled stool samples obtained from five active ocular BD patients and healthy individuals to B10.RIII mice (Ye et al., 2018). Gut microbiome composition in the recipient mice was tested by the ACE index, Shannon index and principle coordination analysis (PCoA) (Supplementary Figure 1 and Figures 1A,B). The PCoA showed that there was a segregation of gut microbiome composition between these two groups. However, we found that there was no statistical significance in ACE index and Shannon index between these groups. After false discovery rate (FDR) correction (FDR < 0.2), gut microbiome composition was different when comparing BD-recipient mice with healthy controls-recipient mice as shown by analysis of the relative abundances of 11 genera and 14 species (Supplementary Tables 3, 4). Up to eight genera and 10 species were enriched in the BD-recipient mice. More importantly, we observed an enrichment in opportunistic pathogen *Parabacteroides* species, sulfate-reducing bacteria (SRB) *Bilophila* species and *Desulfovibrionaceae* species in the BD-treated group. Moreover, there was a reduction in butyrate-producing bacteria (BPB) *Clostridium* species in this group. LDA Effect Size (LEfSe) was used to determine the microbiome most likely to explain differences between these two groups. The results showed 14 genera including *Bilophila* were positively associated with BD-recipient mice, whereas four genera were negatively associated with BD-recipient mice (Figure 1C). Because the human fecal samples used for transplanation in the present study were randomly selected from the samples used in our previous MGS study (Ye et al., 2018), we compared the data of 16S rDNA gene sequence analysis in the present study with those of our previous MGS study. The present results were in line with the data of MGS analysis on Behcet and healthy individuals that *Bilophila* and *Parabacteroides* were enriched in BD patients, whereas the level of *Clostridium* was decreased. Because an aberrant abundance of SRB and BPB was found in the BD-recipient mice, we also investigated the changes of short chain fatty acids (SCFAs) in the stool samples after fecal transplantation by gas chromatography-mass spectrometer analysis (GC-MS). Three kinds of SCFAs including butyric acid, propionic acid and valeric acid concentrations were decreased in the BD-recipient mice (Figure 1D). These data showed that the gut microbiome composition in the BD-recipient mice may contribute to a concomitant SCFA change.

### Dysfunction of the Intestinal Barrier in Mice Following Transplantation of BD Feces

**FIGURE 2:**
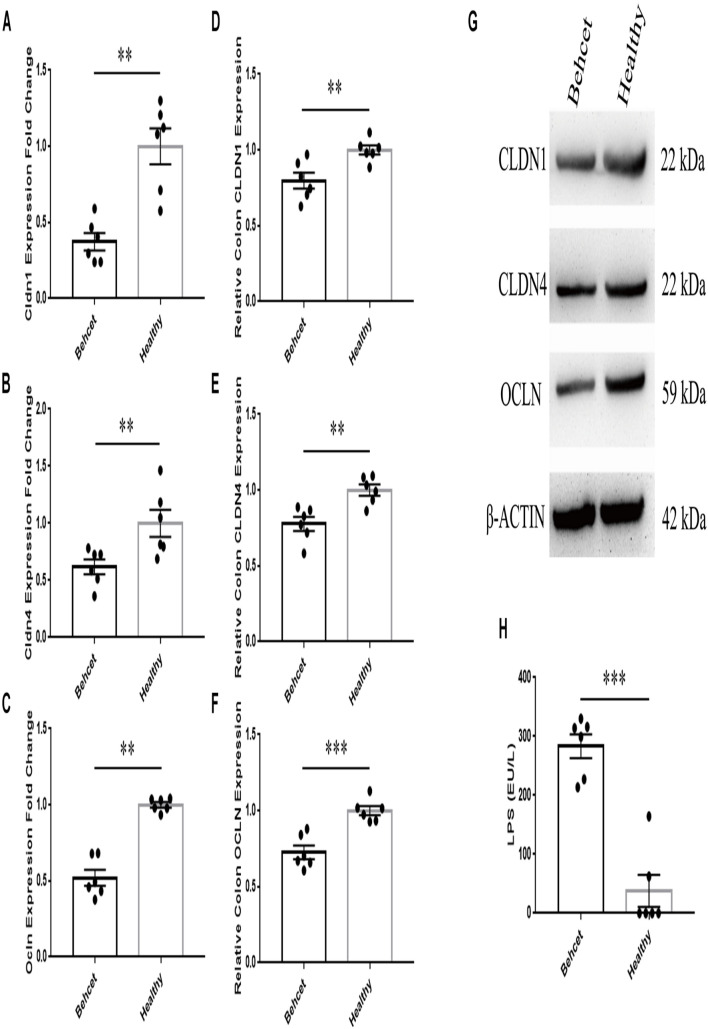
The mRNA and protein expression of tight junction proteins in mouse colon tissue and concentration of LPS in the serum after BD feces transplantation. **(A–C)** Comparison of CLDN1, CLDN4 and OCLN mRNA expression in the colon tissue between BD-recipient and healthy controls-recipient mice. Expression was normalized to β-actin and calculated relative to the healthy control group that was taken as 1.0. ^∗∗^*P* < 0.01. Data was analyzed by the Mann–Whitney *U* test. *n* = 6 for each group; **(D–G)** Comparison of CLDN1, CLDN4 and OCLN protein expression in the colon tissue between BD-recipient and healthy controls-recipient mice, ^∗∗^*P* < 0.01. **(H)** The concentration of LPS in the serum of BD-recipient and healthy controls-recipient mice. ^∗∗∗^*P* < 0.001. Data was analyzed by the Mann–Whitney *U* test. *n* = 6 for each group.

To investigate the effect of BD feces transplantation on intestinal barrier function in mice, we performed the following experiment. Colon tissue was separated from mice after BD feces transplantation and analyzed for mRNA and protein expression. We found that the expression of three tight junction proteins (Epple et al., 2009), Claudin1 (CLDN1), Claudin4 (CLDN4) and Occludin (OCLN) in the colon tissue from the BD-recipient group was significantly decreased as compared to the healthy control-recipient group (Figures 2A–G). We subsequently investigated whether the altered gut microbial composition in the mice following BD feces transplantation could lead to the release of lipopolysaccharides (LPS) into the blood circulation of these animals. Serum was collected from the two groups after gut microbiome transplantation and the results showed that the level of LPS in the serum of the BD-recipient group was significantly increased as compared to that of the healthy control-recipient group (Figure 2H).

### The Effect of Transplantation of BD Feces on Th1 and Th17 Cell Differentiation in Mice

**FIGURE 3:**
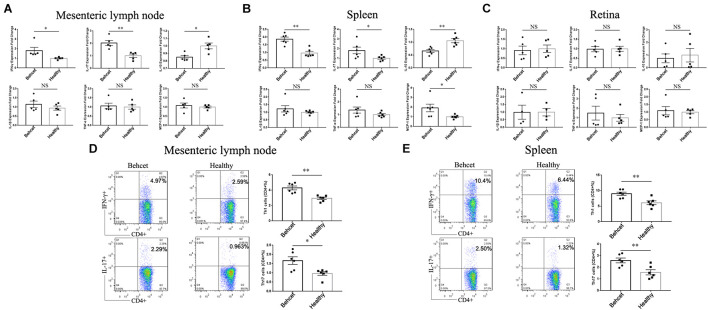
The mRNA expression of IFN-γ, IL-17, IL-10, MCP-1, IL-1β and TNF-α as well as Th1 and Th17 cells in the mesenteric lymph node, splenic lymphocytes and retina after transplantation of BD feces to mice. **(A–C)** Comparison of IFN-γ, IL-17, IL-10, MCP-1, IL-1β and TNF-α mRNA expression in the mesenteric lymph node, splenic lymphocytes and retina between BD-recipient and healthy controls-recipient mice, relative to the healthy control group (taken as 1.0). ^∗∗^*P* < 0.01, ^∗^*P* < 0.05, NS, not significant, data was analyzed by the Mann–Whitney *U* test. *n* = 5–6 for each group; **(D,E)** the percentages of CD4 + IFN-γ + Th1 cells and CD4 + IL-17 + Th17 cells in the mesenteric lymph node and splenic lymphocytes in the BD-recipient and healthy controls-recipient group. ^∗∗^*P* < 0.01, ^∗^*P* < 0.05. Data was analyzed by the Mann–Whitney *U* test. *n* = 6 for each group.

Since BD feces transplantation affected intestinal permeability, we further investigated whether the gut microbiome from BD patients could influence Th1 and Th17 cell differentiation in mice. BD feces was transplanted to mice and on day 7, the animals were sacrificed and mesenteric lymph node, splenic lymphocytes as well as retinal tissue were separated. The IFN-γ and IL-17 mRNA expression was significantly increased in the mesenteric lymph node and spleen of the BD-recipient group as compared with the healthy control-recipient group, whereas IL-10 mRNA expression was decreased in the mesenteric lymph node and spleen (Figures 3A,B). In addition, the mRNA expression of MCP-1 was found to be increased following BD feces transplantation in the splenic lymphocytes (Figure 3B). The transplantation did not lead to ocular inflammation in these non-immunized mice, as shown by real-time PCR analysis of retinal tissue (Figure 3C). The percentages of Th1 cells and Th17 cells in the mesenteric lymph node and splenic lymphocytes of BD-recipient group were higher than that in the healthy control-recipient group (Figures 3D,E). These data indicate that transplantation of the gut microbiome from BD patients to mice induces Th1 and Th17 cell differentiation in the mesenteric lymph node and spleen cells.

### Single Cell RNA Sequencing of Spleen From Mice Following Transplantation of BD Feces

**FIGURE 4:**
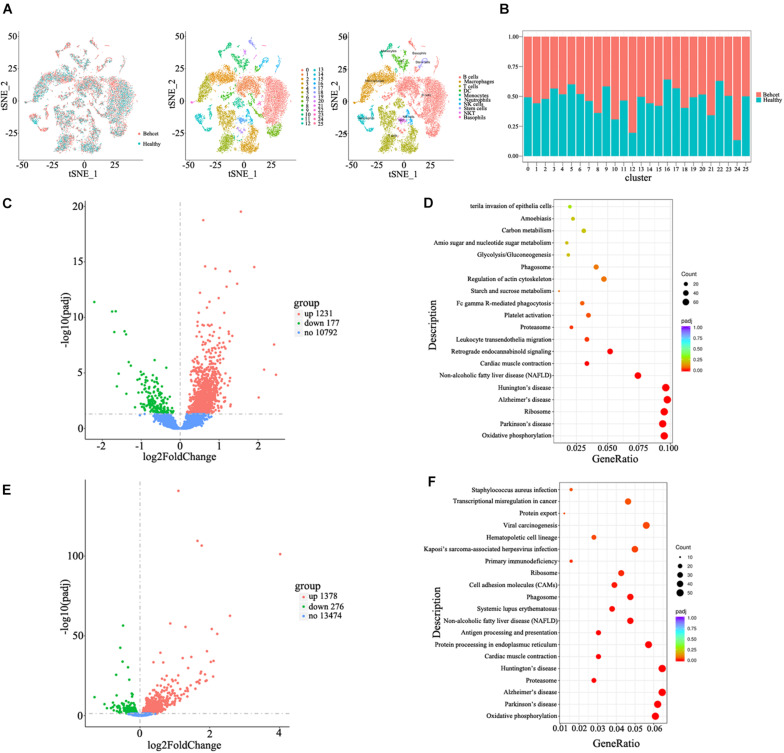
Single cell RNA sequencing of splenic cells from mice after feces transplantation. **(A)** Characterization of cell types in the splenic cells in the tSNE plot; **(B)** The relative ratios of different cell types in the splenic cells between BD-recipient mice and healthy control-recipient mice; **(C)** The volcano plot of differential genes in the neutrophils between BD-recipient and healthy controls-recipient mice; **(D)** KEGG analysis of differential genes in the neutrophils between BD-recipient and healthy controls-recipient mice. Top twenty pathways with the most significant differences are listed; **(E)** The volcano plot of differential genes in the CD4 + T cells between BD-recipient and healthy controls-recipient group; **(F)** KEGG analysis of differential genes in the CD4 + T cells between BD-recipient and healthy controls-recipient mice. Top twenty pathways with the most significant differences are listed.

To investigate the mechanisms underlying the contributions of the gut microbiome from BD patients to the induction of Th1 and Th17 cell differentiation, we performed single cell RNA sequencing of splenic cells from mice after transplantation of BD feces. Twenty six cell clusters were characterized as 10 cell types including B cells, macrophages, T cells, DC, monocytes, neutrophils, NK cells, stem cells, NKT cells and basophils after annotation (Figure 4A). The ratio of neutrophils (cluster 12) was increased in BD-recipient mice as compared to healthy control-recipient mice (Figure 4B). After comparing the mRNA expression levels of neutrophils between these two groups, the expression of 1,321 genes was increased in the BD-recipient mice, whereas 177 genes were decreased (Figure 4C). More importantly, the mRNA expression of Mpo and Elane, the components of NETs, were increased in the BD-recipient mice. In addition, the mRNA expression of S100a8 and S100a9, two antimicrobial proteins, were also significantly increased in the BD-recipient mice (Supplementary Table 5). Functional enrichment analysis of these differential mRNAs *via* KEGG showed bacterial invasion of epithelial cells and leukocyte transendothelial migration was significantly up-regulated in the neutrophils from BD-recipient mice (Figure 4D and Supplementary Table 6). Based on GO enrichment analysis, these differential mRNAs were enriched in the ATP metabolic process, oxidative phosphorylation and several terms associated with neutrophils function, such as neutrophil chemotaxis and neutrophil mediated immunity (Supplementary Figure 2 and Supplementary Table 7).

We also compared the mRNA expression of CD4 + T cells from T cell populations between BD-recipient and healthy control-recipient mice. The expression of 1,378 genes was increased in the BD-recipient mice, whereas 276 genes were decreased (Figure 4E). Among them, IL-17 mRNA expression was significantly increased in the CD4 + T cells from BD-recipient animals (Supplementary Table 8), which was consistent with the results mentioned before. Through KEGG analysis, these differential mRNAs were found enriched in antigen processing and presentation and in Systemic Lupus Erythematosus (SLE, Figure 4F and Supplementary Table 9). GO enrichment analysis was also performed on these differentially expressed mRNAs. The results showed that the mRNAs were enriched in the innate immune response, positive regulation of immune response, T cell activation, lymphocyte differentiation and response to bacteria (Supplementary Figure 3 and Supplementary Table 10).

### Neutrophil Activation Following Fecal Transplantation and the Role of Activated Neutrophils on Th1 and Th17 Differentiation

**FIGURE 5:**
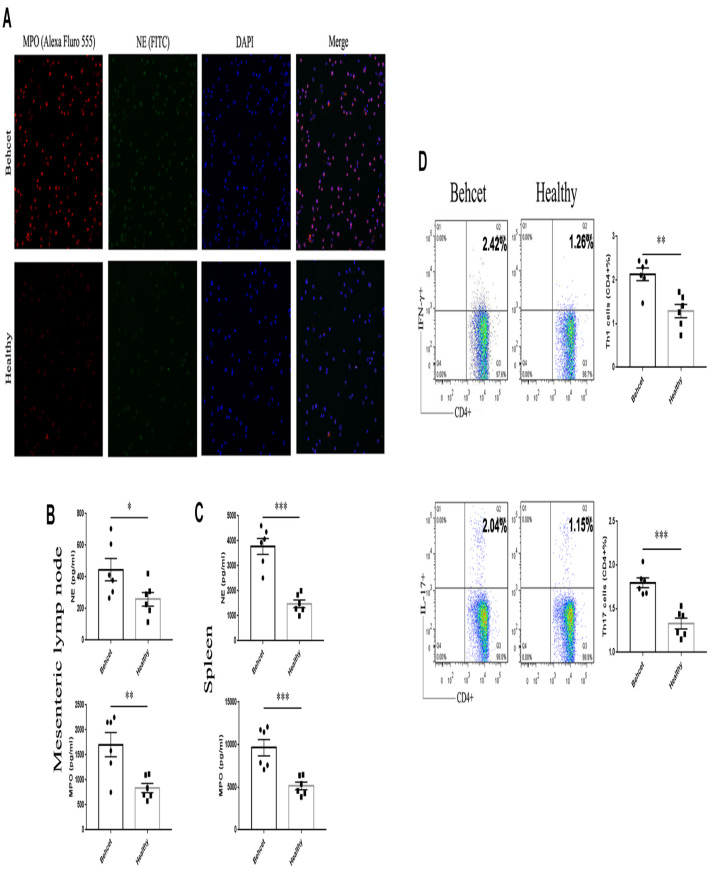
NETs secretion as well as Th1 and Th17 cell differentiation after co-culture with neutrophils. **(A)** Fluorescent images of NETs (MPO and NE) in the BD-recipient group and healthy controls-recipient group. **(B,C)** The protein levels of NE and MPO in the supernatants of mesenteric lymph nodes and splenic neutrophils from the BD-recipient and healthy controls-recipient group. ^∗∗∗^*P* < 0.001, ^∗∗^*P* < 0.01, ^∗^*P* < 0.05. Data was analyzed by the Mann–Whitney *U* test. *n* = 6 for each group. **(D)** the percentages of CD4 + IFN-γ + Th1 cells and CD4 + IL-17 + Th17 cells after co-culture with neutrophil supernatants from the BD-recipient and healthy controls-recipient group. ^∗∗∗^*P* < 0.001, ^∗^*P* < 0.01, NS, not significant. Data was analyzed by the Mann–Whitney *U* test. *n* = 5–6 for each group.

Neutrophils are found to be hyper-activated in patients with BD (Nelson et al., 2018). Numerous factors from the gut microbiome are found to induce neutrophil activation, including LPS (Alexis et al., 2001). Based on the above findings, we hypothesized that the activation of neutrophils in BD patients could be associated with the composition of their gut microbiome. To investigate whether neutrophils can be activated after fecal transplantation, we isolated the total cells from mesenteric lymph nodes and neutrophils from spleens of mice and detected the level of NETs secreted by activated neutrophils after fecal transplantation. After immunostaining neutrophils, we found that NE and MPO were more prominently expressed by neutrophils from the BD-recipient group (Figure 5A). The levels of the NETs components, NE and MPO were significantly increased in the cells from mesenteric lymph nodes and neutrophils from spleens from the BD-recipient group as compared to the healthy control-recipient group (Figures 5B,C). We also co-cultured lymphocytes the mesenteric lymph nodes of normal B10.RIII mice with neutrophil supernatants of the BD-recipient or healthy control-recipient group, respectively. The percentages of Th1 cells and Th17 cells in the BD-recipient neutrophils co-culture group were higher than in the healthy control-recipient neutrophil co-culture group (Figure 5D). These results indicate that the gut microbiome in BD patients might induce Th1 and Th17 cell differentiation *via* activated neutrophils.

### The Effect of Fecal Transplantation With BD Patients Gut Microbiome on the Severity of EAU and EAE Models

**FIGURE 6:**
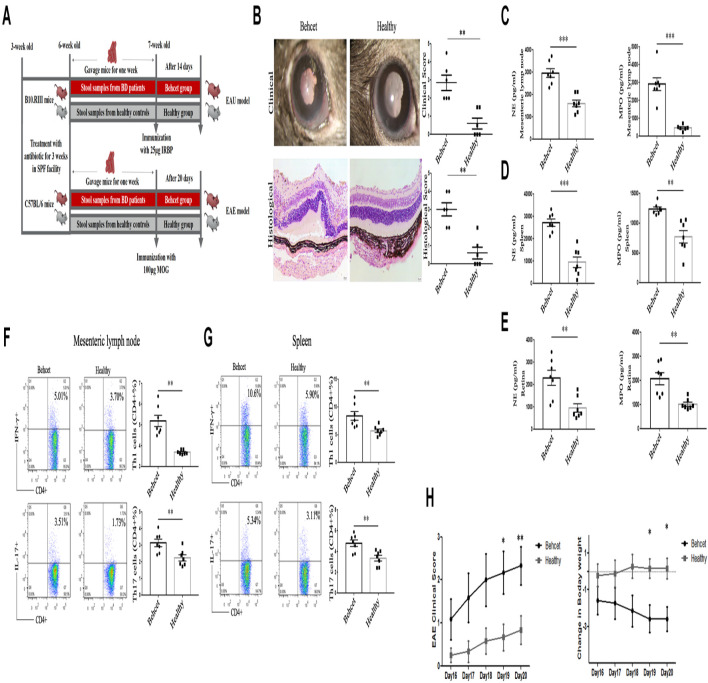
Clinical and histological scores of EAU mice as well as clinical score and weight changes of EAE mice in the BD-recipient and healthy controls-recipient group. **(A)** The experimental scheme of mice experiment; **(B)** Clinical and histological scores of the EAU model in the BD-recipient and healthy controls-recipient group. ^∗∗^*P* < 0.01. Data was analyzed by the Mann–Whitney *U* test. *n* = 6 for each group; **(C–E)** The protein levels of NE and MPO in the supernatants of mesenteric lymph nodes, splenic neutrophils and retina from the BD-recipient and healthy controls-recipient group. ^∗∗∗^*P* < 0.001, ^∗∗^*P* < 0.01. Data was analyzed by the Mann–Whitney *U* test. *n* = 6–7 for each group. **(F,G)** The percentages of CD4 + IFN-γ + Th1 cells and CD4 + IL-17 + Th17 cells in the mesenteric lymph node and splenic lymphocytes from the BD-recipient and healthy controls-recipient group. ^∗∗^*P* < 0.01, ^∗^*P* < 0.05. Data was analyzed by the Mann–Whitney *U* test. *n* = 6–7 for each group. **(H)** Clinical score and weight changes of EAE mice in the BD-recipient and healthy controls-recipient group. ^∗∗^*P* < 0.01, ^∗^*P* < 0.05. Data was analyzed by the Mann–Whitney *U* test. *n* = 6 for each group.

In our previous study, we demonstrated that feces from active BD patients could exacerbate the development and severity of uveitis in EAU mice (Ye et al., 2018). In this study, we repeated the experiment concerning the effect of BD patients’ gut microbiome on EAU and expanded these earlier studies by also analyzing the cytokine response and lymphocyte differentiation. B10.RIII mice were immunized with 25 μg IRBP161–180 combined with CFA for EAU induction after fecal transplantation (Figure 6A). At 14 days after immunization, BD-recipient mice showed a more severe clinical and histological uveitis than the healthy control-recipient group (Figure 6B). In line with these observations, we found that the IFN-γ and IL-17 mRNA expression was also significantly increased in the mesenteric lymph node, splenic lymphocytes and retinas of the BD-recipient group as compared with the healthy control-recipient group (Supplementary Figures 2A,B,D). IL-10 mRNA expression was decreased in the splenic lymphocytes of the BD-recipient group but not in the mesenteric lymph nodes and retina (Supplementary Figures 2A,B,D). The mRNA expression of MCP-1, IL-1β and TNF-α was also increased in the BD-recipient group (Supplementary Figures 2A,B,D). The protein levels of IFN-γ and IL-17 in the splenic lymphocytes of the BD-recipient group were also higher than that in the healthy control-recipient group (Supplementary Figure 2C). We then investigated the activation of neutrophils, Th1 and Th17 cells differentiation in the mesenteric lymph node, spleen and eye between these two groups. In line with the results mentioned before, NE and MPO were significantly increased in the neutrophils from the mesenteric lymph node, splenic lymphocytes and eyes of the BD-recipient group as compared to the healthy control-recipient group (Figures 6C–E). The increased Th1 and Th17 cell percentages were also found in the mesenteric lymph node, spleen and eyes of the BD-recipient group (Figures 6F,G and Supplementary Figure 2E).

To investigate whether the effect of BD patient gut microbiome composition was a general effect or whether it was specific for EAU, we also tested fecal transplantation in mice undergoing EAE (Figure 6A). We therefore immunized C57/BL6 mice with 100 μg Myelin Oligodendrocyte Glycoprotein (MOG) 35–55 combined with CFA after BD feces transplantation to determine its effect on the severity of EAE. The results showed that the BD-recipient mice also showed a more severe clinical manifestation that began at day 19 following immunization (Figure 6H). The expression of pro-inflammatory cytokines, including IFN-γ, IL-17 and MCP-1 was increased in the lymphocytes from the BD-recipient group, whereas the expression of IL-10 was decreased (Supplementary Figures 3A–F). A similar result was also observed in the BD-recipient group concerning the protein expression of IFN-γ and IL-17 (Supplementary Figures 3G,H). These results demonstrated that BD patients’ feces not only exacerbated the severity of EAU but also of EAE, suggesting an immunological adjuvant effect.

## Discussion

**FIGURE 7:**
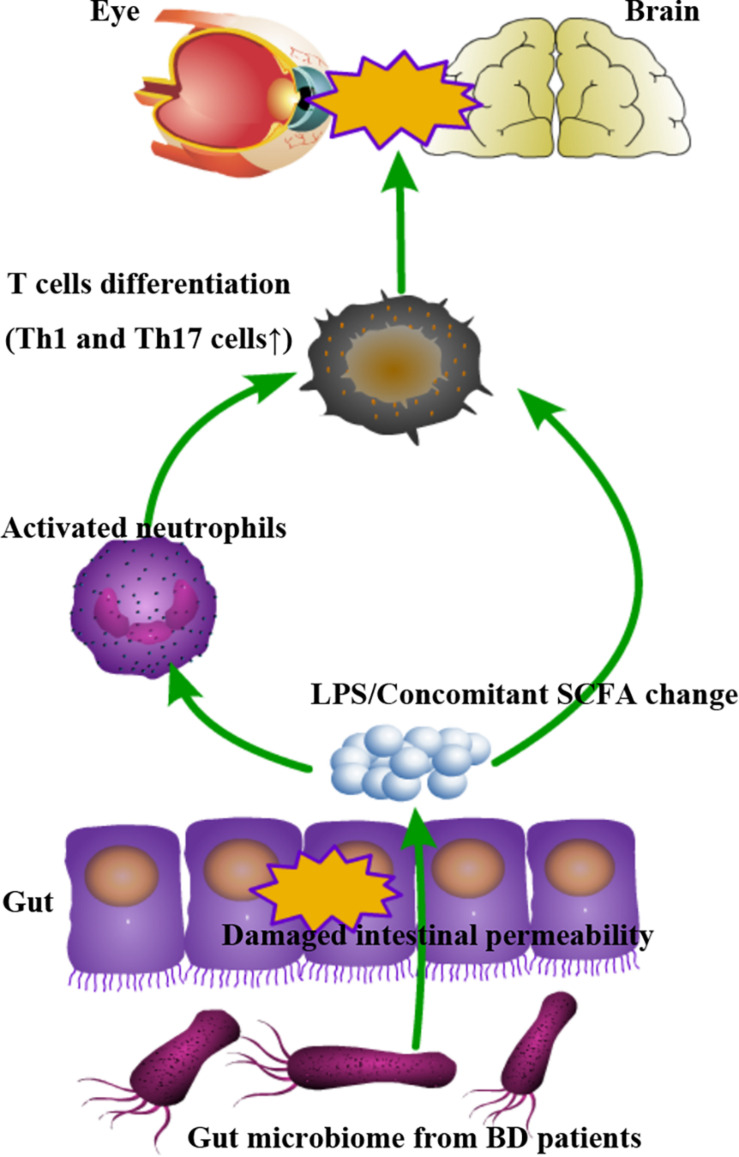
Schematic model demonstrating how the gut microbiome may contribute to the autoimmune response *via* complex mechanisms involving a damaged intestinal barrier leading to the transfer of immuno-stimulatory factors resulting in a state of immune hyper-reactivity.

In this report, we provide evidence showing that the gut microbiome may affect the development of BD *via* a complex mechanism that includes an enhanced gut permeability and stimulation of innate immunity (Figure 7).

Transplantation of clinical fecal samples to mice provides evidence for a role of the gut microbiome in the pathogenesis of various diseases which are found to be associated with a dysbiosis of gut microbiota (De Palma et al., 2017). The differential abundance of bacteria between the BD-recipient group and healthy control-recipient group were not exactly in line with the data of MGS analysis showed in our previous study, which might be due to differences between humans and mice in their indigenous microbial community, the limited sample size and batch effects of microbiome sequencing (De Palma et al., 2017; Ye et al., 2018). It might also be due to the fact that we used SPF mice in this study, although we did treat mice with antibiotics for 3 weeks before fecal transplantation. Using germ-free mice, in future experiments, may solve the issue. On the other hand, we did find that the *Parabacteroides* species, *Bilophila* species and *Desulfovibrionaceae* species were enriched and *Clostridium* species were reduced in the BD-treated group which was in line with our earlier results on the gut microbiome in ocular BD (Ye et al., 2018). *Parabacteroides* species are generally considered as opportunistic pathogens in infectious diseases and are associated with changes in the inflammatory response, T cell differentiation and altered abundance of short-chain fatty acid producers (Claesson et al., 2012; Bindels et al., 2016). *Bilophila* species and *Desulfovibrionaceae* species are two kinds of SRB which inhibit butyrate β-oxidation and degrade butyrate (Dostal Webster et al., 2019). A lower level of BPB such as the *Clostridium* species in the BD-treated group might lead to a reduction of butyrate in the host. Butyrate is a beneficial metabolite that maintains host immune homeostasis and protects the integrity of the intestinal epithelial barrier (Furusawa et al., 2013; Geirnaert et al., 2017; Goncalves et al., 2018). Furthermore, SRB produce cytotoxic molecules such as hydrogen sulfide (H_2_S) as well as immune stimulating factors like LPS (Muyzer and Stams, 2008). These factors can exacerbate intestinal epithelial barrier damage and produce innate immune triggering signals finally leading to an aberrant Th1 and Th17 cell differentiation (Muyzer and Stams, 2008; Leal Rojas et al., 2017; Stevens et al., 2018). Recently, propionic acid and valeric acid (decreased in the BD-treated group) were also reported as SCFAs that can regulate the function of Th1 and Th17 cells (Bottcher et al., 2000; Duscha et al., 2020). We hypothesize that the concomitant SCFA change caused by the gut microbiome from patients may contribute to the development of autoimmune uveitis.

Using the fecal transplantation model, we showed that it could affect intestinal permeability leading to the leakage of bacterial factors such as LPS into the systemic circulation. Although intestinal manifestations are common (3–60%) in BD patients from Eastern Asia (Ananthakrishnan, 2015), it should be noted that none of the ocular BD patients that donated their stool samples for our study had obvious intestinal problems. Intestinal barrier dysfunction has been observed in several immune-mediated diseases such as ankylosing spondylitis (AS), whereas these patients did not have obvious intestinal problems or intestinal inflammation (Vaile et al., 1999). Intestinal barrier function in ocular BD patients has not yet been investigated and is indeed an interesting area for further research. LPS is one of the most potent gram-negative derived factors that can stimulate Th1 and Th17 cell differentiation and is able to exacerbate experimental autoimmune disease models such as EAU and EAE (Fang et al., 2010; Klaska et al., 2017; Mardiguian et al., 2017). These findings are also consistent with previous studies in which LPS/TLR4 pathways are involved in the aberrant immune response observed in BD and our result showing a higher abundance of SRB (such as *Bilophila* species) in the BD-recipient mice (Liang et al., 2013). Whether other microbe-associated molecular patterns induced by the gut microbiome may also contribute to Th1 and Th17 differentiation needs to be addressed in future experiments. It should be noted that the specificity of the Th1 and Th17 cells in the BD-recipient mice was not investigated and it would be interesting to study whether feces transplantation affected the population of autoreactive cells. However, the increase of Th1 and Th17 cells in the BD-recipient mice might lead to an augmented secretion of pro-inflammatory cytokines including IFN-γ, TNF- α, IL-6 and IL-17 that may contribute to the development of disease (Tang et al., 2009; Pattarini et al., 2017).

Previous studies have implied a role for infections in the pathogenesis of BD, including streptococcus species (Lellouche et al., 2003; Oh et al., 2008). In the study presented here, we provide additional evidence that gut microbiota from BD patients can induce innate immune responses and that this response might play a role in the stimulation of pathogenic T cell populations. An alternative mechanism is also possible where IL-17 triggers neutrophil production and release from the bone marrow (Chuammitri et al., 2019). IL-17 has been shown to recruit neutrophils at the site of T cell activation by inducing the production of chemokines and other cytokines, such as TNF-α (Isailovic et al., 2015). Further studies are needed to show how Th17 cells and neutrophils interact and how this is affected by gut microbiota. The results of single cell sequencing on splenic cells from mice after fecal transplantation also provided evidence that the gut microbiome from BD patients has effects on both neutrophil activation as well as on T cell differentiation. We found that the differential mRNAs in neutrophils were enriched in the oxidative phosphorylation, neutrophil chemotaxis and neutrophil mediated immunity, which all play critical roles in the activation of neutrophils (Li et al., 2019). When analyzing CD4 + T cells, we found that the IL-17 mRNA level was increased in CD4 + T cells from BD-recipient mice. Differentially expressed mRNAs in CD4 + T cells were found enriched in antigen processing and presentation. It is well established that antigen processing and presentation induces activation and differentiation of naïve T cells into Th1 and Th17 cells (Mascanfroni et al., 2013). These results indicate that the gut microbiome in BD-recipient mice might contribute to both neutrophil activation and regulation of T cell differentiation. It should also be mentioned that other cell clusters, such as cluster 24 in B cells, appear to be distinct between BD and healthy-recipient mice. It would be interesting to study the contributions of these cell clusters to disease in future studies. To investigate whether the gut microbiome regulates the function of neutrophils or CD4 + T cells directly, single cell sequencing analysis on intestinal tissue or MLN from mice and human blood is also needed to address this issue in future longitudinal studies.

To our knowledge, this is the first report that directly links a dysbiotic gut microbiome to the activation of neutrophils in BD. There is evidence in the literature that the link between neutrophil activation and the induction of Th1 and Th17 cell differentiation may be mediated by dendritic cells (Warnatsch et al., 2015; Papadaki et al., 2016). Taken together these findings support our hypothesis that a combination of gut metabolites and immune-stimulatory factors behave as an adjuvant leading to a hyperactive immune system.

We realize that our study suffers from several limitations concerning the protocol of fecal transplantation. First of all, we didn’t perform a comparison between recipient mice and original donors in the present manuscript due to sample size limitations in the original donors used for transplantation. To further validate our study, further longitudinal studies with a larger sample size are needed to address this issue. Secondly, fecal transplantation was done with pooled patient or healthy control fecal samples in the present study. Gut microbiome diversity could be very different between individuals. Thus, the pooled samples may not be a faithful representation of a real-world individual gut microbiome composition. Fecal transplantation using individual samples may be a favorable method in future experiments. In addition, the donors included in this study had stopped taking immunosuppressive and antibiotic treatment for at least 1 month. However, several studies have suggested that past treatment, such as with antibiotics, may have contributed to the observed changes in the gut microbiome composition of host (Palleja et al., 2018). Using samples from the first-episode BD patients without treatment may solve this issue in future studies. Thirdly, the immunization in the mice may also affect the gut microbiome (Janowitz et al., 2019). To evaluate whether immunization has an influence on the transplanted microbiome, analysis of microbiome composition should be performed before and after immunization.

In conclusion, we show that the gut microbiome may contribute to the development of BD *via* complex mechanisms involving a damaged intestinal barrier leading to the transfer of immuno-stimulatory factors resulting in a state of immune hyper-reactivity. The observations shown in this paper are focused on the eye but similar mechanisms may be operative in other autoimmune diseases and manipulation of the gut microbiome may be a feasible approach to treat these disorders.

## Data Availability Statement

The original contributions presented in the study are publicly available in NCBI using accession number PRJNA741894.

## Ethics Statement

The animal study was reviewed and approved by the animal study was approved by the Ethics Committee of The First Affiliated Hospital of Chongqing Medical University.

## Author Contributions

QW and PY conceived and directed the study. QW, SY, GS, ZD, SP, and XH analyzed the data. PY made the clinical diagnoses. QC collected the samples. QW and XH extracted the fecal DNA and performed the 16S rRNA gene amplicon sequencing, *in vitro* experiment, and animal experiment. GY evaluated histology slides. QW drafted the manuscript. PY and AK reviewed the data interpretation and helped revise the final versions of the manuscript. All authors read and approved the final manuscript.

## Conflict of Interest

The authors declare that the research was conducted in the absence of any commercial or financial relationships that could be construed as a potential conflict of interest.

## Publisher’s Note

All claims expressed in this article are solely those of the authors and do not necessarily represent those of their affiliated organizations, or those of the publisher, the editors and the reviewers. Any product that may be evaluated in this article, or claim that may be made by its manufacturer, is not guaranteed or endorsed by the publisher.

## Footnotes

1
https://support.10xgenomics.com/single-cell gene expression/software/pipelines/latest/what-is-cell-ranger

2
https://github.com/dviraran/SingleR

3
https://satijalab.org/seurat/

## Supplementary Material

Supplementary Figure 1 ACE index analysis results comparing the BD-recipient and healthy controls-recipient group.

Supplementary Figure 2 The mRNA expression of IFN-γ, IL-17, IL-10, MCP-1, IL-1β and TNF-α from mesenteric lymph node, splenic neutrophils and retina, the protein levels of IFN-γ and IL-17 from splenic lymphocytes as well as Th1 and Th17 cells differentiation from the retina in the EAU models from BD-recipient and healthy controls-recipient mice. **(A,B)** Comparison of IFN-γ, IL-17, IL-10, MCP-1, IL-1β and TNF-α mRNA expression from mesenteric lymph nodes, splenic neutrophils and retina in the EAU models in BD-recipient and healthy controls-recipient mice, ^∗∗∗^*P* < 0.001, ^∗∗^*P* < 0.01, ^∗^*P* < 0.05, NS, not significant. Data was analyzed by the Mann–Whitney *U* test. *n* = 6 for each group; **(C)** IRBP161-180-induced production of IFN-γ and IL-17 at the protein level in the BD-recipient and healthy controls-recipient group. ^∗∗∗^*P* < 0.001, Data was analyzed by the Mann–Whitney *U* test. *n* = 6 for each group. **(D)** Comparison of IFN-γ, IL-17, IL-10, MCP-1, IL-1β and TNF-α mRNA expression from the retina of EAU mice between the BD-recipient and healthy controls-recipient group, ^∗∗^*P* < 0.01, ^∗^*P* < 0.05, NS, not significant. **(E)** The percentages of CD4 + IFN-γ + Th1 cells and CD4 + IL-17 + Th17 cells in the retina from the BD-recipient and healthy controls-recipient group.^∗^*P* < 0.05. About 2,000–4,000 CD4 + T cells from the eye were collected from the retina for each sample. Retinas of 2 mice from each group were pooled as one sample. Data was analyzed by the Mann–Whitney *U* test. *n* = 4 for each group (eight mice).

Supplementary Figure 3 Go analysis of differential genes in the neutrophils between BD-recipient and healthy controls-recipient mice. Top ten terms with the most significant differences are listed. MF, molecular function; BP, biological process; CC, cellular component.

Supplementary Figure 4 Go analysis of differential genes in the CD4 + T cells between BD-recipient and healthy controls-recipient mice. Top ten terms with the most significant differences are listed. MF, molecular function; BP, biological process; CC, cellular component.

Supplementary Figure 5 The mRNA expression of IFN-γ, IL-17, IL-10, MCP-1, IL-1β and TNF-α as well as the protein levels of IFN-γ and IL-17 from splenic lymphocytes in the EAE mice of the BD-recipient and healthy controls-recipient group. **(A–F)** Comparison of IFN-γ, IL-17, IL-10, MCP-1, IL-1β and TNF-α mRNA expression in the EAE mice of the BD-recipient and healthy controls-recipient group, ^∗∗^*P* < 0.01, ^∗^*P* < 0.05, NS, no significant. Data was analyzed by the Mann–Whitney *U* test. *n* = 6 for each group; **(G,H)** IRBP161-180-induced production of IFN-γ and IL-17 at the protein level in the BD-recipient and healthy controls-recipient group. ^∗^*P* < 0.05, Data was analyzed by the Mann–Whitney *U* test. *n* = 6 for each group.
