# Supplementary figures and images for: Changes in the Gut Microbiome Contribute to the Development of Behcet’s Disease via Adjuvant Effects
Source: Front Cell Dev Biol. 2021 Sep 8;9:716760. doi: 10.3389/fcell.2021.716760 (PMC8455896; doi:10.3389/fcell.2021.716760)

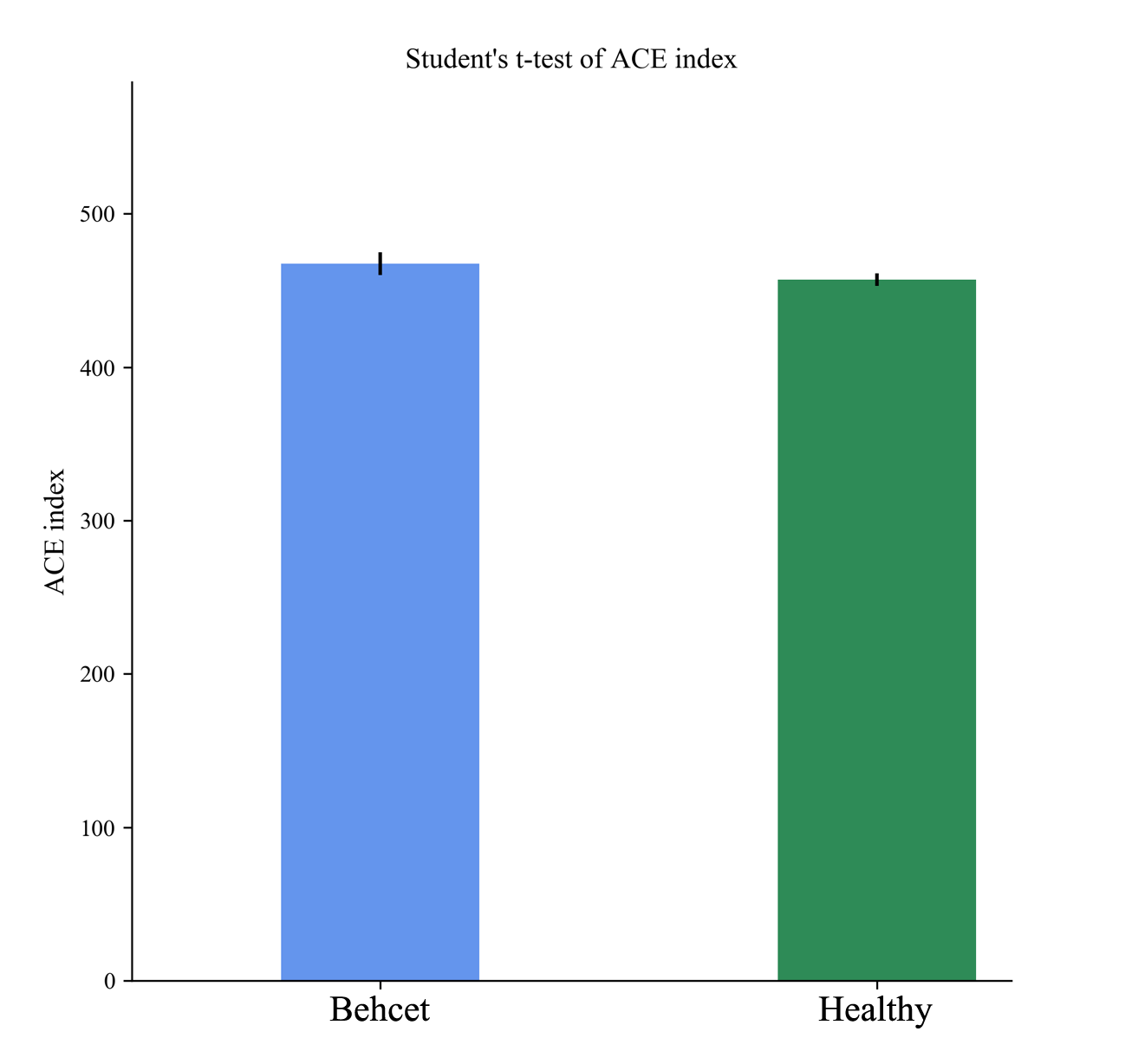

Supplement: Supplementary Figure 1 — ACE index analysis results comparing the BD-recipient and healthy controls-recipient group. [file Image_1.TIF]

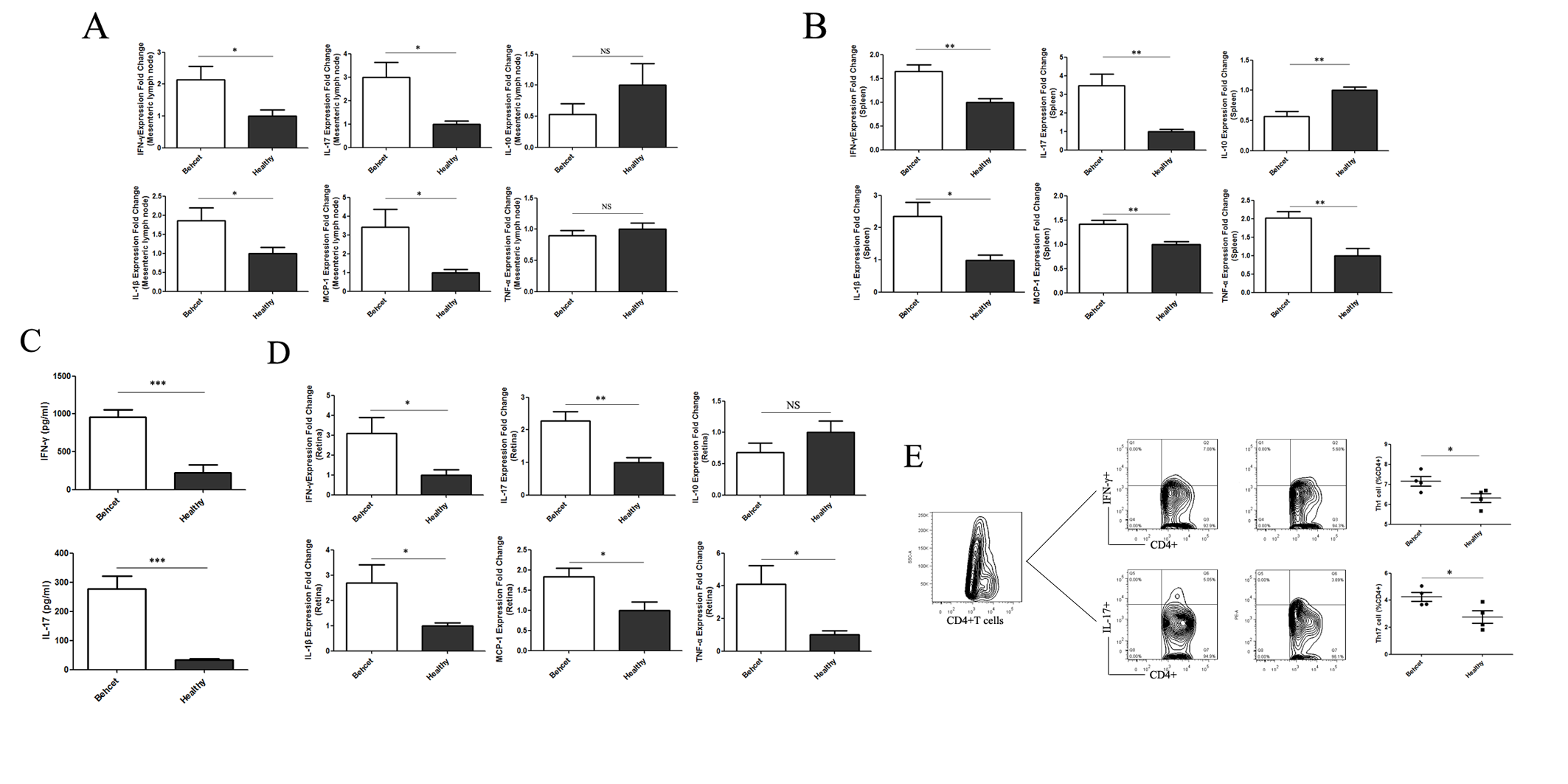

Supplement: Supplementary Figure 4 — Go analysis of differential genes in the CD4 + T cells between BD-recipient and healthy controls-recipient mice. Top ten terms with the most significant differences are listed. MF, molecular function; BP, biological process; CC, cellular component. [file Image_4.TIF]

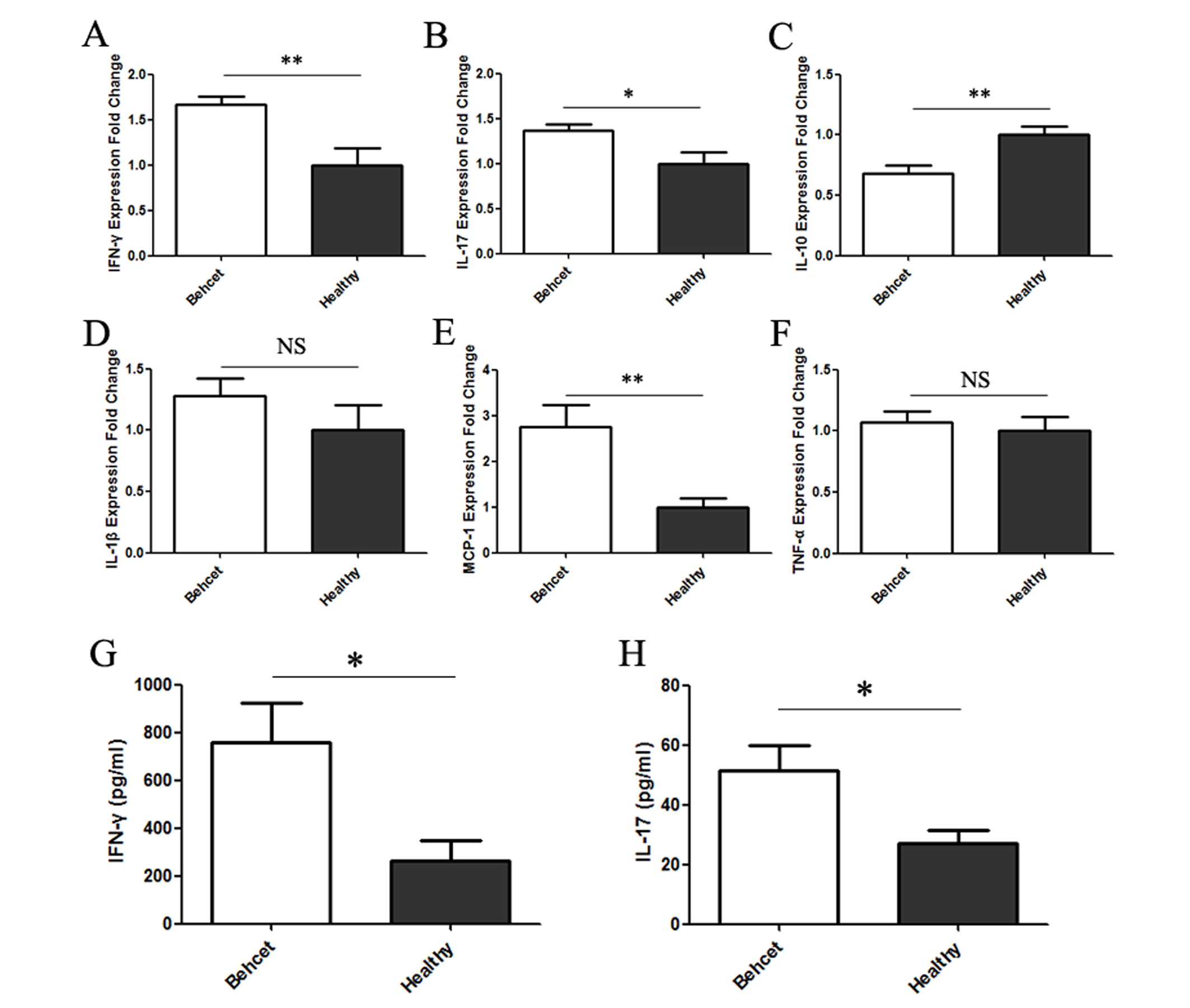

Supplement: Supplementary Figure 5 — The mRNA expression of IFN-γ, IL-17, IL-10, MCP-1, IL-1β and TNF-α as well as the protein levels of IFN-γ and IL-17 from splenic lymphocytes in the EAE mice of the BD-recipient and healthy controls-recipient group. (A–F) Comparison of IFN-γ, IL-17, IL-10, MCP-1, IL-1β and TNF-α mRNA expression in the EAE mice of the BD-recipient and healthy controls-recipient group, ∗∗P < 0.01, ∗P < 0.05, NS, no significant. Data was analyzed by the Mann–Whitney U test. n = 6 for each group; (G,H) IRBP161-180-induced production of IFN-γ and IL-17 at the protein level in the BD-recipient and healthy controls-recipient group. ∗P < 0.05, Data was analyzed by the Mann–Whitney U test. n = 6 for each group. [file Image_5.TIF]
